# Assessment and aesthetic impact of a long‐term vertical discrepancy between the single anterior maxillary implant‐supported crown and adjacent teeth: A retrospective cross‐sectional study

**DOI:** 10.1002/cre2.629

**Published:** 2022-08-27

**Authors:** Grégoire Sauvin, Nathalie Nurdin, Mark Bischof, Stavros Kiliaridis

**Affiliations:** ^1^ Division of Orthodontics University Clinics of Dental Medicine Geneva Switzerland; ^2^ Swiss Dental Clinics Group Ardentis Clinique Dentaire Lausanne Lausanne Switzerland; ^3^ Department of Orthodontics and Dentofacial Orthopedics University of Bern Bern Switzerland

**Keywords:** infraocclusion, single maxillary anterior implant, smile satisfaction, vertical changes

## Abstract

**Objectives:**

To assess the vertical discrepancy between implant‐supported crowns and adjacent teeth in the maxillary anterior region at least 8 years after implant placement and to evaluate the influence of this discrepancy on the level of aesthetic awareness of patients.

**Material and Methods:**

The sample consisted of 23 adult individuals evaluated at least 8 years after placement of an implant‐supported central or lateral single tooth‐fixed partial denture. Patients had their crowns delivered at a mean age of 47.8 years (range: 18.9–65.8). The vertical discrepancy was measured by comparing initial and follow‐up periapical radiographs using the implant as a stable structure. The patients' satisfaction with their anterior teeth condition and awareness of the possible vertical problem were evaluated using a questionnaire. The aesthetic outcome and patient awareness were related to the objective measurement of the vertical discrepancy.

**Results:**

The implant showed a mean infraocclusion of 0.62 mm (range: 0.15–1.63 mm). The vertical discrepancy was not associated with the patient's gender, age at implant placement, and duration between initial and recall radiograph. Patients were generally satisfied with the long‐term aesthetic outcome of their smile (mean: 3.9 on a 1–5 scale, 1 unsatisfied, and 5 completely satisfied). Out of 23 patients, 8 noticed the implant infraocclusion and 4 of them found the problem severe enough to be willing to improve the situation. The amount of vertical discrepancy was not associated with the patient's perception of the discrepancy and the pink aesthetic score.

**Conclusion:**

Implant‐supported crowns in the anterior region may suffer infraocclusion over the long term. The amount of vertical discrepancy was not dependent on the gender and age of the patient. Patients were generally satisfied with the aesthetic result of the restoration. The amount of vertical discrepancy, at least in the range we have measured, was not perceived by the patients as a complication.

## 1 INTRODUCTION

In adults, the replacement of a missing anterior maxillary permanent tooth is often performed using an implant‐supported crown. This treatment is not performed in growing patients because of the infraocclusion occurring at the implant site due to the vertical development of the alveolar process and the continuous eruption of the adjacent teeth (Johansson et al., 1994; Odman et al., 1991; Thilander et al., 2001). Thilander et al. (2001) recommended delaying the treatment of young patients until the end of their growth or to consider an alternative treatment such as adhesive fixed partial bridges. However, it has been shown by Bernard et al. (2004) that despite the recommendation mentioned above, the development of infraocclusion of an implant‐supported crown might occur in adults. A vertical discrepancy may develop in both young and mature adult subjects 4 years after the placement of the implant. Similar results were reported by Vilhjálmsson et al. (2013) who also observed that female patients showed a higher risk than male subjects of developing infraposition of the implant‐supported crowns with respect to the adjacent teeth. The infraposition of the implant‐supported crowns was also observed by Schwartz‐Arad and Bichacho (2015) who claimed that the older adults presented a minor degree compared to the younger adults.

As shown by Christou and Kiliaridis (2008), large variability among individuals was reported, which might be due to the variation in the amount of secondary growth. Facial morphology and the intraocclusal forces may regulate the amount of continuous eruption (Kiliaridis et al., 2019; Winitsky et al., 2021).

Despite the huge number of implants that have been placed over the last three decades, and quite a few of them in the anterior maxillary region as single implants, the impact of the vertical discrepancy on the patients' aesthetic perception of their smile some years after the implant placement was not reported.

The aim of this retrospective observational study was to quantitatively assess the vertical discrepancy between the implant‐supported crowns and adjacent teeth in the anterior region in patients treated for at least 8 years. Furthermore, the aesthetic impact of this discrepancy was evaluated as well as the patients' awareness of this situation.

## 2 MATERIAL AND METHODS

### 2.1 Subjects

The database of private clinics (Ardentis Clinique Dentaire) was searched for patients treated with a single implant in the anterior maxillary region at least 8 years after implant placement.

The inclusion criteria were the following: adult patients with an anterior central or lateral maxillary incisor implant‐supported crown for at least 8 years; the presence of the adjacent teeth for control; no active pathology (chronic periodontal disease, peri‐implantitis); no replacement of the initial implant‐supported crown and no abnormal wear of the contralateral tooth.

The sample size calculation was performed using Stata/IC 16.1 and was based on the information of the primary outcome, that is, the vertical discrepancy between the implant‐supported crown and adjacent anterior maxillary teeth, as obtained from a pilot study we performed on 10 subjects. The standard deviation in the vertical displacement in this pilot sample was 0.4 mm. The calculation was based on the assumption that 0.3 mm vertical discrepancy is the least clinical significant difference in the subjects treated with an implant‐supported crown to replace an anterior maxillary tooth. A sample size of 21 patients was expected to have 90% power (Power (1−*ß*) = 0.9) to detect this 0.3 mm difference between the initial and the follow‐up periapical radiographs using the paired *t*‐test, with a 0.05 two‐sided significance level (Alpha (*α*) = 0.05). To compensate for possible imperfections in the measurement procedure, we increased the sample by 10%. Thus, the total sample was planned to be 23 subjects.

To reach this number, a total of 81 patients were contacted by phone or email. Twenty‐nine patients could not be reached, nine were not willing to participate, eight were excluded because of orthodontic treatment, restoration of the adjacent teeth or crown replacement, two had relocated, and one passed away. Thirty‐two patients accepted a complimentary examination in Ardentis Clinique Dentaire.

Previous clinical data (initial and intermediate radiographs, photographs, and periodontal assessments) and information on crown replacement were available from the patients' medical files. From the initial sample, nine patients were excluded because of missing initial apical radiograph taken immediately after crown placement, leading to the final sample of 23 individuals required for the study. The dental implants received by the included patients (Straumann AG, Basel, Switzerland) were placed by two experienced oral surgeons. Six implants were placed immediately after extraction of the tooth and in 12 cases, a GBR (guided bone regeneration) was performed using autogenous bone graft (three cases) and xenogenous bone graft (nine cases).

This study was approved by the Ethics Committees of the University Hospitals of Lausanne (Switzerland) for human research under protocol reference number 2017‐01986. Informed consent was obtained from every subject before entering the study.

### 2.2 Clinical examination

A periodontal assessment of the maxillary anterior region was performed. It included probing depth and bleeding index to exclude individuals with chronic periodontal disease or peri‐implantitis.

The pink and white aesthetic score as modified by Belser et al. (2009) (PES/WES) was used to assess the aesthetics of the implant restoration several years after implant placement. The five gingival parameters were assessed from 0 (worst outcome) to 2 (best outcome). The maximal possible score was 10.

### 2.3 Patient satisfaction and awareness

A questionnaire was used to evaluate the perception of the patients on their maxillary anterior teeth condition (Table 1). General questions concerning their satisfaction with the aspect of their smile were asked as well as details on aesthetical characteristics like color, shape, position, and height of the teeth/gingiva. The questionnaire was mainly composed of questions from previous studies evaluating the aesthetic outcome and the patient satisfaction of individuals with anterior implant‐supported crowns (Belser et al., 2008; Graber & Lucker, 1980; Vilhjálmsson et al., 2011). The patients were also asked how they perceived the changes in these features (color, shape, position, height) over time. Questions 1 to 7 were answered on a 1 to 5 scale for which 1 was considered being very unsatisfied and 5 very satisfied whereas questions 8 to 11 were binary yes/no questions.

**Table 1:** Patient satisfaction and awareness questionnaire

Questions	Answer	
(Q1) How do you feel about the appearance of your teeth?	Very unsatisfied—totally satisfied 1 2 3 4 5	
(Q2) Have you found that other people have commented negatively on the appearance of your teeth?	Very often—never 1 2 3 4 5	
(Q3) How do you perceive the appearance of your teeth compared to others?	1. Uglier than everybody else 2. Uglier than most 3. About the same kind of teeth as everybody else 4. Prettier than most 5. Prettier than everybody else	
(Q4) Concerning the shape of your anterior teeth you are…	1. Very unsatisfied 2. Unsatisfied 3. Neutral 4. Satisfied 5. Very satisfied	
(Q5) Concerning the color of your anterior teeth you are…	1. Very unsatisfied 2. Unsatisfied 3. Neutral 4. Satisfied 5. Very satisfied	
(Q6) From an aesthetic point of view, how do you feel about the treatment…	1. Very unsatisfied 2. Unsatisfied 3. Neutral 4. Satisfied 5. Very satisfied	
(Q7) Concerning the general aspect of the gingiva around the implant‐supported crown, you are…	1. Very unsatisfied 2. Unsatisfied 3. Neutral 4. Satisfied 5. Very satisfied	
(Q8) If you could change something to your anterior upper teeth, would you change…	a. Color	Yes–No
	b. Shape	Yes–No
	c. Position	Yes–No
	d. Height	Yes–No
	e. Gingiva	Yes–No
(Q9) Did you notice changes in the general aspects of your teeth during time?	Yes	
	No	
(Q10) Did you notice changes in the height of your teeth during time?	Yes	
	No	
(Q11) Did you notice changes in the height of your gingiva during time?	Yes	
	No	

### 2.4 Radiological assessment

A periapical radiograph of the implant and the adjacent teeth was taken using the “Parallel technique” with an “X‐Mind AC” or an “X‐Mind System Image X” Satelec® machine.

The radiograph taken immediately after the implant restoration (baseline T0) found in the file of the patient and the radiograph taken at the time of control (T1) were printed with high quality and 10 times magnification.

The internal calibration of each radiograph was done by comparing the radiographic implant size with that given by the manufacturer. If the implant was not completely visible on the radiograph, the interthread distance defined by the manufacturer was used as a reference.

**Figure 1:**
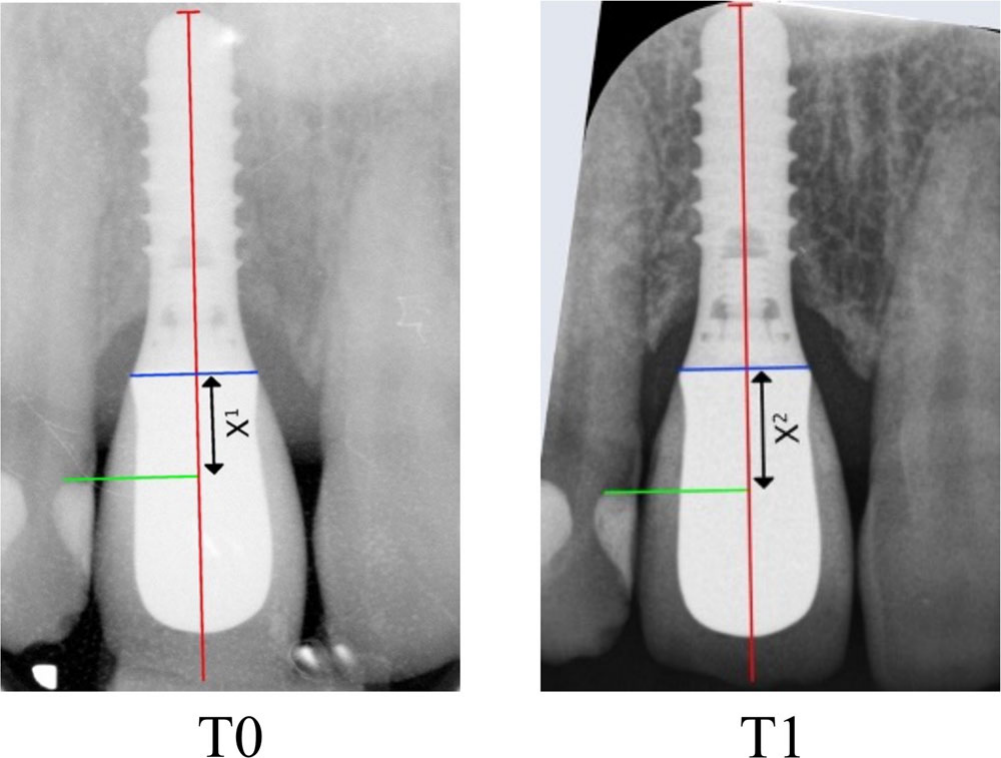
Radiograph taken at prosthesis placement (reference T0) and at the long‐term control (T1). The implant length was measured on each radiograph and compared to that given by the manufacturer for internal calibration. Reproducible landmark (green) on the adjacent teeth was selected on T0 and T1 radiographs. The projection of the T0 landmark (green) on the long axis of the implant only (red) and not the crown established the baseline distance X1. The same measurement was made on the T1 image. X2 was then subtracted to X1 to determine the amount of vertical discrepancy between T0 and T1.

An assessment of the eruption was performed using the implant as a stable structure (Figure 1). Reproducible landmarks on the adjacent teeth were selected on T0 and T1 images. If no clear landmarks were found on one adjacent tooth, this tooth was excluded to minimize error. The distance between the projection of the landmark on the implant's long axis (not the crown) and the implant's neck was measured. The difference in this value measured on T0 and T1 radiographs expressed the discrepancy between the implant‐supported crown and adjacent teeth.

### 2.5 Statistics

The primary outcome concerning the vertical discrepancy between the implant‐supported crown and adjacent anterior maxillary teeth was evaluated with a paired *t*‐test calculation between the initial and the follow‐up radiograph. Linear and logistic regression analyses were used to test the impact of the discrepancy and the objective aesthetic score (PES/WES) on the patients' awareness of this situation as perceived by the questionnaire. The level of statistical significance was set at 5%.

The error of the method was calculated by measuring twice, 1 month apart, the vertical discrepancy between implants and adjacent teeth in 15 randomly selected cases. The random error was calculated using the Dahlberg formula (Dahlberg, 1940) Se = $\sqrt{\frac{\Sigma d^{2}}{2n}}$ = 0.118 mm. No systematic error could be detected using paired *t*‐test. A high correlation was found between the repeated measurements (*r*: 0.79).

## 3 RESULTS

Twenty‐three patients (13 females and 10 males) were included in the study. Implants were positioned in the central position in 12 cases and in the lateral position in 11 cases. The mean age of patients at implant placement was 47.8 years (range: 18.9–65.8 years). The average duration between implant restoration (T0) and control (T1) was 12.5 years (range: 8.0–17.1 years). The characteristic of each patient is presented in Table 2.

**Table 2:** Sample description, implant position, follow‐up period, and mean vertical discrepancy between the implant‐supported crown and the adjacent tooth

Subject	Sex	Age at implant placement (years)	Central (1) lateral (2)	Follow‐up period (years)	Mean discrepancy (mm)
1	Male	18.8	2	17.1	0.57
2	Male	38.6	1	12.9	0.46
3	Male	21.1	1	13.6	0.57
4	Male	51.9	1	13.8	0.44
5	Male	56.6	1	11.6	0.44
6	Male	26.9	2	12.0	1.48
7	Male	63.5	2	10.6	0.47
8	Male	35.5	2	14.7	0.62
9	Male	65.8	1	7.9	0.31
10	Male	49.9	1	11.4	0.73
11	Female	45.6	1	11.3	0.79
12	Female	58.6	2	12.0	0.47
13	Female	44.6	2	16.9	0.57
14	Female	51.9	2	12.9	0.60
15	Female	63.2	2	12.6	0.28
16	Female	46.3	1	14.2	0.79
17	Female	51.5	1	9.0	0.45
18	Female	55.0	2	13.6	0.15
19	Female	60.2	1	8.9	0.24
20	Female	54.0	2	10.4	0.44
21	Female	45.0	2	12.8	0.87
22	Female	47.1	1	14.5	1.63
23	Female	47.6	1	11.6	0.90

All studied implants showed a mean infraocclusion of 0.62 mm (SD: 0.35 mm) varying from 0.15 to 1.63 mm. The amount of vertical discrepancy was associated neither to the patients age at implant placement (*R* = −0.11; *p* ≤ .62) nor to the duration between baseline and control radiographs (*R* = 0.10; *p* ≤ .66). No difference in infraocclusion was found between males (mean: 0.61 mm; SD: 0.33) and females (mean: 0.63 mm; SD: 0.39) and between central (mean: 0.65; SD: 0.37) and lateral (mean: 0.59; SD: 0.35) incisors regarding the amount of vertical discrepancy.

Questionnaires were fulfilled by all the included patients (Table 3). Patients were generally satisfied with the long‐term aesthetic outcome of their smile (Q1, mean: 3.9) and more specifically with the implant‐supported crown (Q6, mean: 3.9). To a lesser extent, they estimated the aesthetic of their smile “about the same as everybody else” (Q3, mean: 3.0). Furthermore, patients were less satisfied with the color of their anterior teeth (Q5, mean: 3.1).

**Table 3:** Patient satisfaction and awareness questionnaire: Answer distribution

Questions	1	2	3	4	5
(Q1) How do you feel about the appearance of your teeth? *Very unsatisfied (1)–Totally satisfied (5)*	2	2	4	3	12
(Q2) Have you found that other person has commented negatively on the appearance of your teeth? *Very often (5)–Never (1)*	12	8	2	1	0
(Q3) How do you perceive the appearance of your teeth compared to others? *Uglier than everybody else (1)–Prettier than everybody else (5)*	0	5	14	4	0
(Q4) Concerning the shape of your anterior teeth you are? *Very unsatisfied (1)–Totally satisfied (5)*	2	10	9	2	0
(Q5) Concerning the color of your anterior teeth you are? *Very unsatisfied (1)–Totally satisfied (5)*	2	5	9	7	0
(Q6) From an aesthetic point of view how do you feel about the treatment? *Very unsatisfied (1)–Totally satisfied (5)*	0	0	5	15	3
(Q7) Concerning the general aspect of the gingiva around the implant‐supported crown you are? *Very unsatisfied (1)–Totally satisfied (5)*	4	5	10	3	1

The gingival aesthetics were satisfactory for the patients. They were generally pleased with the overall aspect of their gingiva (Q7, mean: 3.3). Seven of them had noticed changes in height of the gingiva during time Q11 (30%). At the follow‐up evaluation, implants showed a mean PES score of 6.69 (SD: 1.7) and a WES of 7.61 (SD: 1.3)

When the 23 patients were asked if they had noticed general changes in the anterior region during time (Q9), 11 of them (48%) answered affirmatively. Eight patients (35%) found that the vertical position of the anterior teeth had changed (Q10) and four (17%) would like to correct the vertical discrepancy of their anterior teeth (Q8d.).

After implementing linear and logistic regression analysis to test the impact of the PES/WES on the questionnaire's answers, the only statistically significant observation concerned the PES and the patient's awareness of a vertical change of its gingiva around the implant. Patients who had a high measured PES had a lower odds ratio (OR) to notice the vertical change of the gingiva surrounding the implant (Q11) OR = 0.48, CI: 0.25–0.96, *p* = .039.

The patients who noticed the vertical discrepancy in their maxillary anterior region were not those who presented the higher value measured on radiographs.

The sex and age of subjects were tested as a predictor of the perception by the patient of the vertical discrepancy, but the results did not reveal any statistical significance.

## 4 DISCUSSION

The present study has shown that all patients included in this study and followed for 12.5 years on average presented a vertical discrepancy between the implant‐supported crown and adjacent teeth in the maxillary anterior region. The mean value of this discrepancy was 0.62 mm with a large range between the individuals (0.15–1.63 mm). Our results are in line with studies by Bernard et al. (2004) and Vilhjálmsson et al. (2013), and also with a recent meta‐analysis performed by Papageorgiou et al. (2018) who calculated a mean implant infraposition of 0.58 mm (range: 0.33–0.85 mm).

Neither the age nor the gender of patients influenced the value of the vertical discrepancy. Literature concerning the difference between males and females is controversial; Jemt et al. (2007) and Andersson et al. (2013) found that females had a higher risk of having more severe implant infraocclusion than males whereas Bernard et al. (2004), Winitsky et al. (2021), and Brahem et al. (2017) did not find any differences. However, the studies cited above had relatively small sample sizes. In a meta‐analysis based on these studies, Papageorgiou et al. (2018) found that females had a higher risk of infraocclusion.

In the present study, the patients' age at implant placement was not associated with the severity of the infraocclusion. Similar results were found by Andersson et al. (2013), Chang and Wennström (2012), Cocchetto et al. (2019), and Bernard et al. (2004). In a previously published study, Schwartz‐Arad and Bichacho (2015) showed a difference in the severity of the infraocclusion between a young group (<30 years) and a more mature group (>30 years) but the measurements were done solely on the final photographs and were not measured on the absolute vertical discrepancy but evaluated as a percentage of the crown size.

In our sample, the length of the observation period does not seem to influence the amount of vertical discrepancy, possibly due to the small number of cases with big variation among individuals.

In the past, it was thought that the placing of an implant in adult patients was stable as soon as growth had stopped. After that, multiple studies have shown that remaining vertical growth occurred (Behrents, 1985; Ghislanzoni et al., 2017; Thilander et al., 1999). This vertical growth in adult subjects was sometimes called secondary growth. The vertical discrepancy that occurs due to the continuous eruption takes place throughout life (Christou & Kiliaridis, 2008) with a big variability. It has been shown that facial morphology and the intraocclusal forces may regulate the amount of continuous eruption (Kiliaridis et al., 2019). Unfortunately, neither evaluation of the facial morphology of the patients nor the functional capacity of the muscles were available for this sample.

Individuals with implants in the anterior upper region were generally satisfied with the treatment result at the long‐term follow‐up. It has been shown that most of them were satisfied with the long‐term result (Q1, mean: 3.9) and considered their smile aesthetically pleasing compared to the general population despite the replaced tooth. Previous studies found similar results concerning long‐term satisfaction (Derks et al., 2015; Pjetursson et al., 2005; Simonis et al., 2010). In a systematic review regrouping 11 articles, Arunyanak et al. (2017) found that patients were satisfied with a mean range score of 43%–93% for peri‐implant soft tissue and 81%–96% for implant restorations. Multiple studies showed that implant restoration was rated more satisfactory than peri‐implant mucosa (Cosyn et al., 2012; den Hartog et al., 2013; Meijndert et al., 2007). Our results fall in the same range and proportion, patients were also more pleased with the implant restoration (Q6, mean: 3.9) than the soft tissue (Q7, mean: 3.3). The subjective aesthetic evaluation made by the patients was confirmed by the calculated PES/WES. The general PES at long‐term evaluation was acceptable (mean: 6.69, SD: 1.7). A previous study (Belser et al., 2009) found a mean of 7.8 ± 0.8 but the follow‐up period was shorter (2–4 years), which may explain the difference in our results. In our study, the PES/WES was evaluated by an orthodontist, who has been shown to be clearly more critical than other professional observers (Fürhauser et al., 2005). In our sample, the patients who had noticed a change in the height of their gingiva during time were those that had significantly lower PES scores.

**Figure 2:**
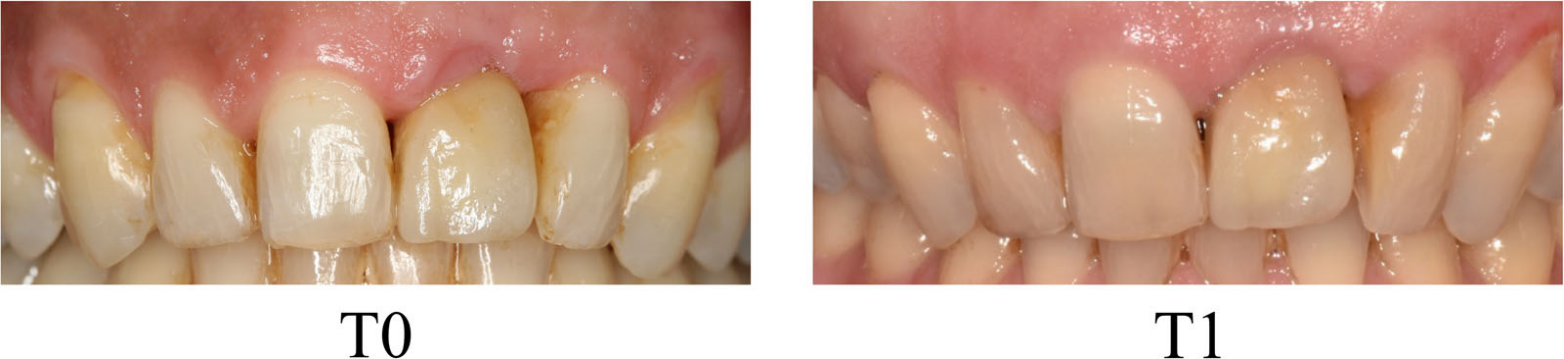
Female patient with a single implant‐supported crown replacing the 21 with an important vertical discrepancy (0.9 mm) after an 11‐year follow‐up who is fully satisfied with the aesthetic outcome of the treatment and did not perceive any changes during time.

Interestingly, the measured severity of the vertical discrepancy was not correlated with the perception of a less aesthetic smile. These results are in line with recent findings by Winitsky et al. (2021) who found no differences in VAS scores between groups of patients with severe (>1 mm) and less severe (<1 mm) infraposition. This big variability in awareness among individuals is illustrated in Figure 2. The patient presented a big vertical discrepancy (0.9 mm) and was not aware of the vertical problem while other subjects with less vertical discrepancy were displeased with their situation. Since the problem develops gradually, changes may not be evident at first. When the patients were asked more specifically if they had noticed vertical changes during time, 35% found that the vertical position of the anterior teeth had changed and 17% wished to change this vertical mismatch. These results are in line with the findings of Cocchetto et al. (2019) who observed very similar results (29% of awareness and 18% of patients who could be seeking retreatment). The relatively low awareness of patients to the vertical changes, even in cases with an important infraocclusion of the implant‐supported crown, can be explained because the true extrusion of the adjacent teeth is measured parallel to the implant. However, the perception of this infraocclusion for the patient is less severe as the changes measured radiographically are visualized in the vertical plane.

Multiple factors are considered in the evaluation of patients and clinician satisfaction with the anterior aesthetics of single implants. The weight of each of them may vary significantly between patients and professionals. The patients are generally less critical than dental professionals (Hartlev et al., 2014; Palmer et al., 2007). The understanding of the patients' thoughts is the key point. Therefore, recent studies are trying to develop and validate new reliable aesthetic indexes that are better associated with the patient's consideration (Li et al., 2019).

In the present study, the age at implant placement can predict neither a higher patient satisfaction nor a lower awareness of the vertical discrepancy. These results contrast with those of Derks et al. (2015) who found that older patients perceived in a more positive manner the long‐term results of their implant‐supported restorations (Ghislanzoni et al., 2017). Our results might be underpowered to detect similar findings.

The limitation of this study was the relatively small sample size. Nine patients were not willing to participate in the study; these refusals might introduce a selection bias since they could possibly conceal an outlier.

On the other hand, the strengths were the long‐term follow‐up (12.5 years) and the standardized radiological recordings that permitted measurements of the vertical discrepancy detected on the adjacent teeth.

Even with a vertical discrepancy evolving over time up to 1.63 mm, it does not elicit demands for review or re‐intervention by patients. Nevertheless, general dentists and oral surgeons should inform patients about this potential long‐term complication. Currently, dental research has not identified clear factors predictive of a higher risk for the vertical discrepancy in adult patients.

## 5 CONCLUSION

All studied implant‐supported crowns showed infraocclusion occurring since a mean time of 12.5 years after implant restoration. The mean measured value of the vertical discrepancy was 0.62 mm. The amount of vertical discrepancy was not dependent on gender nor the location of the replaced anterior tooth (central or lateral incisor). Furthermore, the age of patients at implant placement and duration between initial and recall recordings were not associated with the severity of the infraocclusion.

Smile aesthetics were generally satisfactory despite involving an implant‐supported crown in the anterior maxilla. A minority of patients had noticed the implant infraocclusion (35%) and 17% found the aesthetic defect severe enough to be willing to correct it.

## AUTHOR CONTRIBUTIONS

All authors were involved in the conception, design, data acquisition, data analysis, interpretation, drafting of the manuscript, and its critical revision. All authors gave final approval of the version to be published. The study was supervised by Stavros Kiliaridis.

## CONFLICT OF INTEREST

The authors declare no conflict of interest.

## Data Availability

The data that support the findings of this study are available from the corresponding author upon reasonable request.
